# The Effect of 4‐7‐8 Breathing Exercise Technique on Tinnitus Handicap, Psychological Factors, and Sleep Quality in Tinnitus Patients: A Randomized Controlled Study

**DOI:** 10.1002/brb3.70854

**Published:** 2026-02-12

**Authors:** Gulce Kirazli, Suheda Baran, Gokce Saygi Uysal, Aykut Ozdogan, Serpil Mungan Durankaya, Mehmet Fatih Ogut

**Affiliations:** ^1^ Department of Audiology, Faculty of Health Sciences Ege University Izmir Turkey; ^2^ Department of Audiology, Faculty of Health Sciences Bakircay University Izmir Turkey; ^3^ ENT Clinic Ankara Etlik City Hospital Ankara Turkey; ^4^ Audiometry Program, Vocational School of Healthcare Dokuz Eylul University Izmir Turkey; ^5^ Department of ENT, Faculty of Medicine Ege University Izmir Turkey

**Keywords:** 4‐7‐8 breathing exercise, psychological factors, tinnitus

## Abstract

**Objective:**

The aim of our study was to evaluate the effect of the 4‐7‐8 breathing exercise on tinnitus handicap, psychological factors, and sleep quality.

**Methods:**

The present study employed a parallel‐group randomized controlled trial design. A total of 23 patients with subjective tinnitus in the experimental group and 25 patients with subjective tinnitus in the control group took part in the study. Both groups received 1 hour of informative session on tinnitus, and the experimental group also performed 4‐7‐8 breathing exercises for 6 weeks. Visual analog scale (VAS), tinnitus handicap inventory (THI), insomnia severity index (ISI), trait anxiety inventory (TAI), and perceived stress scale‐10 (PSS‐10) were applied before and on the day the 6‐week program is done or 6 weeks after the informative session is completed.

**Results:**

When the experimental group and the control group were compared after the intervention, a significant decrease was found in all questionnaire and VAS scores of the experimental group. While the questionnaire and VAS scores of the control group after the session did not differ significantly from the baseline scores, all questionnaire scores of the experimental group after the 6‐week program showed a significant decrease compared to the pre‐application scores.

**Conclusions:**

This exercise technique can be used as a simple, effective, and supportive therapy method in the clinical management of tinnitus patients.

**Trial Registration:**

ClinicalTrials.gov identifier: NCT06360731

## Graphical abstract

The 4‐7‐8 breathing exercise significantly reduced tinnitus handicap and psychological stress, and improved sleep quality in tinnitus patients. This study highlights the potential of this simple breathing technique as an effective supportive therapy in the clinical management of tinnitus.

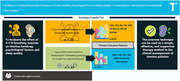

## 1 Introduction

Tinnitus is the perception of sound in the absence of an external sound source (Gilles et al. 2022). The treatment of tinnitus includes medications (ginkgo biloba, antidepressants, anxiolytics, and sedatives), psychological strategies (counseling and cognitive behavioral therapy), auditory methods (tinnitus retraining therapy, sound therapy), and other methods (biofeedback and electromagnetic stimulation) (Apoorva et al. 2023).

Recently, many people with chronic diseases have used complementary therapies to improve their quality of life (Ismail et al. 2022). Yoga is an ancient holistic system originating in India that contains physical positions (asana), meditation (yoga nidra and savasana), and breathing exercises (pranayama) (Köksoy et al. 2018). Complementary therapy Pranayama (“Prana” vital force and “Yama” control/regulation) is originally a Sanskrit term. It is a form of breathing exercise used in yoga that signifies “regulation/control of vital energy” (Ismail et al. 2022). It has been shown that yoga reduces sympathetic nervous system activity, which decreases levels of the stress hormone cortisol. Since yoga is frequently connected to stress and anxiety, it is believed to be beneficial in reducing the intensity of tinnitus (Apoorva et al. 2023).

Based on the yoga technique of pranayama, the 4‐7‐8 breathing exercise aims to facilitate sleep and reduce anxiety. Breathing in by counting to four, holding the breath by counting to seven, and releasing the breath by counting to eight is the method of 4‐7‐8 breath control, developed by American physician Weil A. (Vierra et al. 2022).

Breathing exercises such as the 4‐7‐8 technique require minimal training, no special skills, and can be performed by anyone, anywhere, without the need for equipment or incurring any cost. These exercises are a safe practice and offer a valuable non‐pharmacological alternative or complement to pharmaceutical treatments for addressing mental health issues and other related concerns (Shaw‐Metz 2023).

In this 4‐7‐8 breathing control or breathwork method, short‐term slow breathing reduces oxygen consumption, heart rate (HR), and blood pressure (BP) while increasing the amplitude of theta and delta waves in the brain, thereby accelerating parasympathetic activity and improving the sympathovagal balance in favor of parasympathetic activity (Russo et al. 2017).

This kind of controlled breathing technique can also influence cortical structures that regulate emotions, mood, and arousal. Breathlessness or the anticipation of breathlessness is perceived as threatening and activates limbic structures involved in emotion generation while deactivating cortical structures related to emotional regulation, such as the prefrontal cortex (Banzett et al. 2000).

In the literature, individuals with tinnitus have been found to have higher levels of depression, anxiety, and stress compared to those without tinnitus (Gomaa et al. 2014). Moreover, a review on tinnitus and depression included 20 studies, and 18 of them found a positive correlation between tinnitus and depression (Geocze et al. 2013).

Individuals with high anxiety and panic disorder have less tolerance for breathlessness. Increased activity in the anterior insula, which plays a significant role in the interoception of visceral signals, can be observed in these individuals (Harrison et al. 2021). Controlled breathing exercises can reduce anxiety by decreasing anterior insula activity (Yackle et al. 2017). For instance, Dr. Weil refers to the 4‐7‐8 breathing technique as a “natural calming agent for the nervous system because this technique helps individuals to decrease their anxiety and anger levels and aids in sleep” (Shaw‐Metz 2023).

In a recent systematic review article including five different studies on the effects of Hatha yoga, Bhramari pranayama, pranayama, Ashtanga yoga, and relaxation exercises on tinnitus, a decrease in tinnitus‐related severity, stress, anxiety, and irritability and an increase in life quality were found (Gunjawate and Ravi 2021).

In the literature, there is no randomized controlled study examining the effect of the direct 4‐7‐8 breathing method on tinnitus perception and related psychological variables. Although it is a physiologically and psychologically beneficial, cost‐effective, and safe method, evidence regarding its efficacy in the treatment of tinnitus remains insufficient.

The purpose of our study was to assess the effect of the 4‐7‐8 breathing exercise method on tinnitus severity, tinnitus‐related disability, annoyance, distress, psychological factors, and sleep quality and to compare the results with those of the group just receiving an informative session on tinnitus.

## 2 Materials and Methods

### 2.1 Study Design

This study employed a parallel group randomized controlled trial (RCT) design.

### 2.2 Study Sample and Setting

The study sample consisted of chronic subjective tinnitus patients admitted to the Audiology Unit of the Department of Otorhinolaryngology, Ege University Faculty of Medicine Hospital, and the Otolaryngology Clinic of Etlik City Hospital. The study was conducted between March and June 2024. This study was conducted in accordance with the Helsinki Declaration of 1975 as revised in 2013.

In this study, patients aged 18–65 years with subjective idiopathic tinnitus complaints for at least 6 months, who were not actively receiving any tinnitus treatment or counseling, and who could speak and read Turkish were included. Patients with any neuro‐otologic, psychiatric, neurologic, or cognitive problems; objective tinnitus; chronic medication use; asthma diagnosis or respiratory distress; not answering the questionnaire questions; and not performing the 4‐7‐8 breathing exercises regularly were excluded from the study.

### 2.3 Ethical Considerations

The university's Ethics Committee for Non‐Interventional Clinical Research provided ethical permission for the study (Decision No.: 1018). All participants signed the informed consent form. The trial was registered as an RCT at ClinicalTrials.gov under trial number (NCT06360731).

### 2.4 Sample Size

The G*Power 3.1.9 package program was used to perform a power analysis of the study. Using Cohen's criteria, an effect size of 0.60, a 5% margin of error (alpha), and 90% power, the minimum required sample size was calculated as 40 in total (20 for one group and 20 for other group).

### 2.5 Randomization and Allocation

Nine of the 127 tinnitus patients who were evaluated for study eligibility declined to take part, and 58 of the patients were dropped because they failed to meet the inclusion criteria.

**FIGURE 1:**
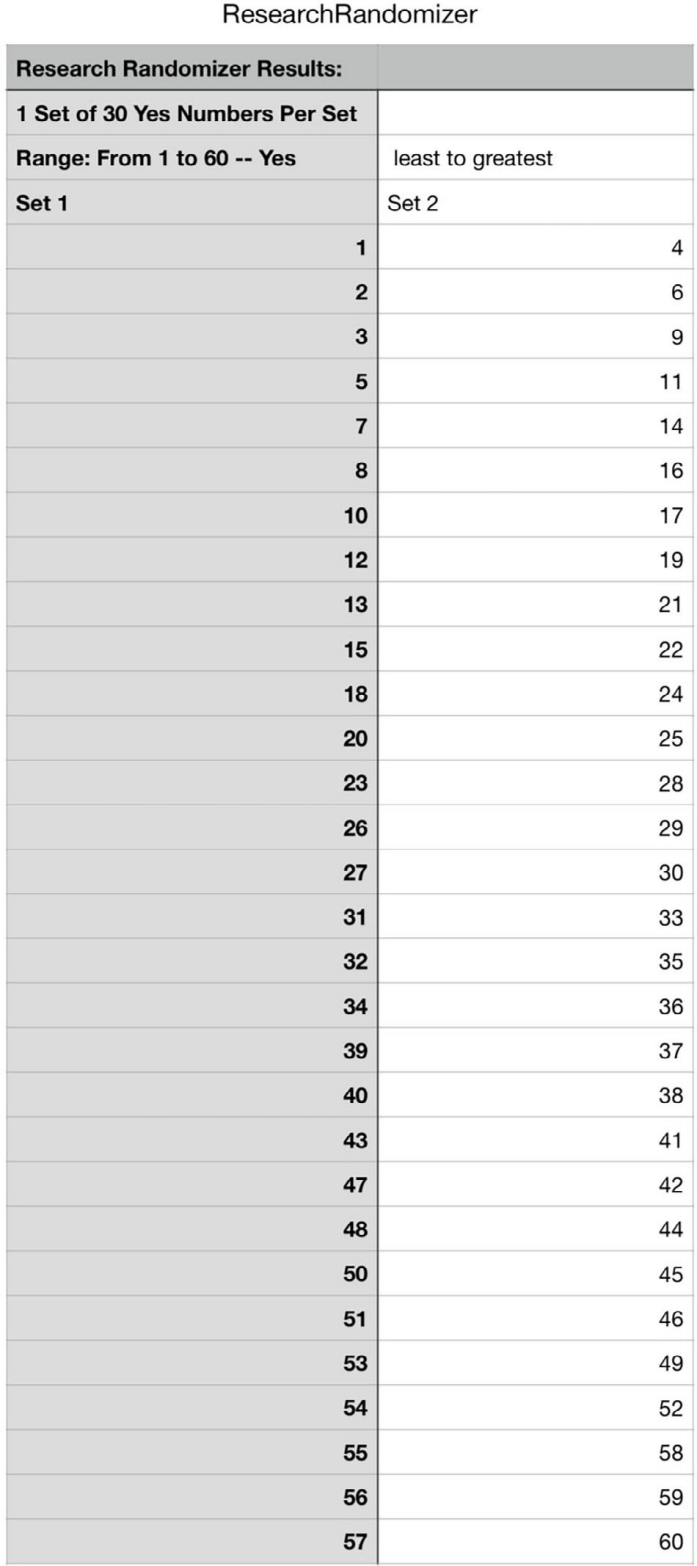
Randomization result table generated by randomizer.org for participant assignment.

A simple randomization method was used by assigning sequential numbers to 60 participants via computer software (https://www.randomizer.org/). The instructions provided on the website https://randomizer.org/ were followed. One set of numbers was selected, containing 30 unique numbers assigned between 1 and 60. For the second set of numbers, the remaining numbers were manually entered. After clicking the “Randomize Now” button, the result table was downloaded. The table is provided in Figure 1.

Maintaining a 1:1 allocation ratio, 30 participants were assigned to the experimental group (patients who received 4‐7‐8 breathing exercises and 1 h of an informative session on tinnitus) and 30 participants were assigned to the control group (the patients who received only 1 h of an informative session on tinnitus) with equal distribution using a simple randomization method. The person conducting this process was a clinical staff member independent of the study, outside the research team.

**FIGURE 2:**
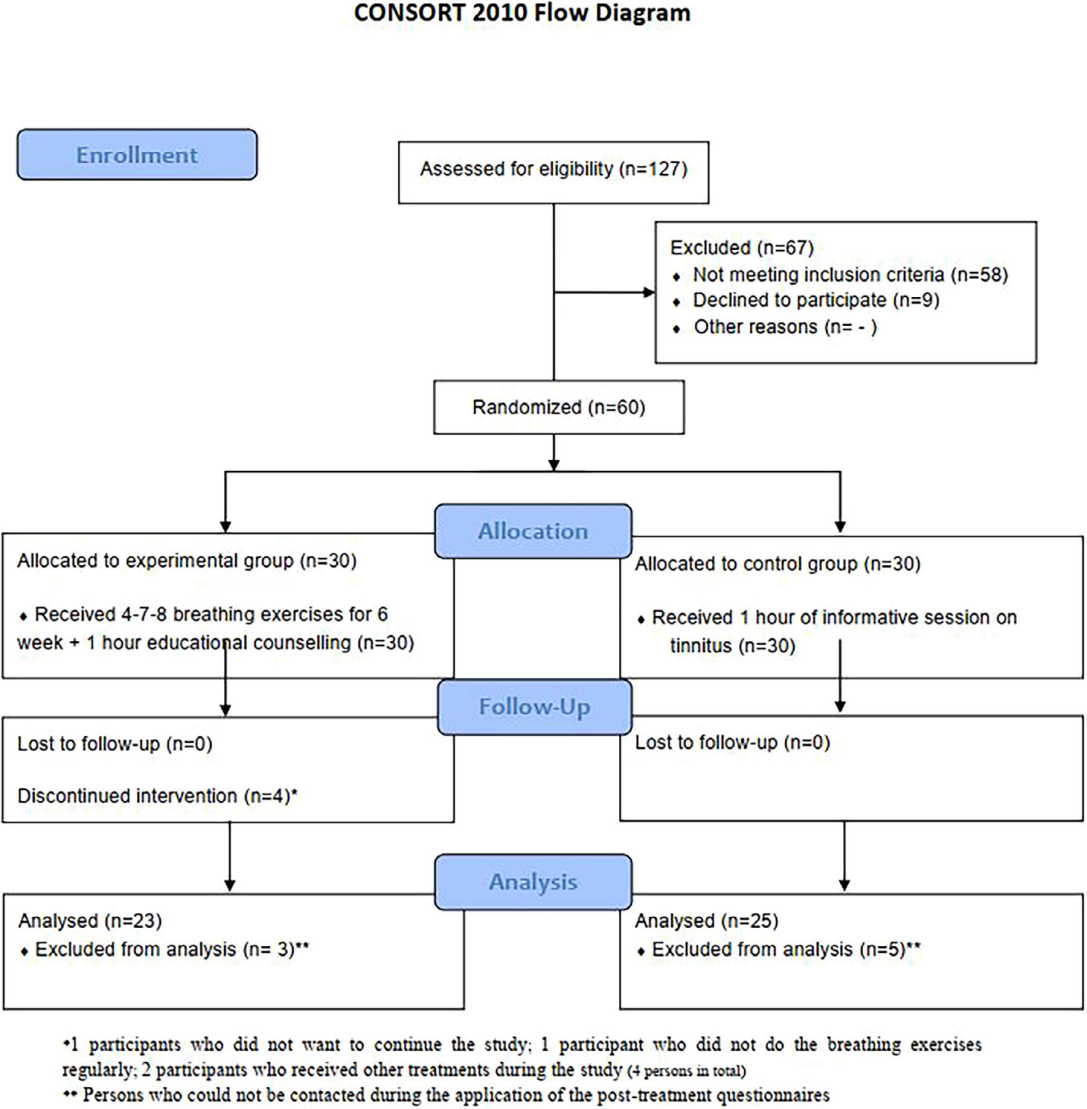
Consort 2010 flow diagram for randomization of groups.

A total of seven participants in the experimental group were excluded from the study, including one participant who did not want to continue the study, one participant who did not do the breathing exercises regularly, and two participants who received other treatments during the study. In addition, three participants from the experimental group and five participants from the control group could not be contacted during the application of the posttreatment questionnaires, so their final evaluations could not be made and were therefore excluded from the study. A total of 48 participants in total enrolled in the study; 23 were in the experimental group and 25 were in the control group. The authors have followed CONSORT for the study (Schulz et al. 2010). The CONSORT 2010 flow diagram is given in Figure 2.

### 2.6 Blinding

In this study, while patients were aware of which group they were assigned to, measures were implemented to minimize potential bias. To prevent bias in the study, blinding was performed between the researchers. The researchers (S.B. and A.O. from both clinics) responsible for data collection were blinded to the participants' group allocations to reduce the risk of observer bias. They made the pre‐ and posttest evaluations without knowing which group the patients were in. Moreover, while participants were informed that one group would perform breathing exercises, we emphasized that the study's goal was to assess different interventions without suggesting that one would necessarily be more effective than the other. This approach aimed to reduce expectation bias among participants. The researchers (G.K. and G.S.U.) administering the breathing intervention and informative session on tinnitus were not involved in data collection to minimize any unintentional influence on participants or results.

### 2.7 Interventions

Following their random assignment to either the experimental or control group, the experimental group underwent 1 h of informative session on tinnitus in addition to 4‐7‐8 breathing exercises, whereas the control group was provided with 1 h of informative session on tinnitus alone. The study was conducted in two centers.

Participants in both groups were invited to the clinics. Participants in the experimental group were individually provided with informative session on tinnitus for 1 h by researchers G.K. and G.S.U. from both clinics. During the informative session, the participants were given general information about tinnitus and were informed about the possible causes, symptoms, diagnosis, therapy, treatment processes, and coping methods. The researchers G.K. and G.S.U. informed each patient by making a presentation they prepared together, which included the abovementioned topics, during the informative session. In addition to the presentation prepared for the informative session to be standard, the same researchers also wrote the sentences together that they would say in the presentation in advance and expressed them in the same way, and conveyed them to the patients. A printed same informative brochure on tinnitus was given to each participant.

In the same session, to ensure uniformity, all participants were provided with detailed, standardized written instructions and demonstration videos on how to perform the 4‐7‐8 breathing exercises after 1 h of informative session. The same exercise protocol was followed by participants. Furthermore, the researchers G.S.U. and G.K. provided the initial training session and were available for questions throughout the intervention period. This ensured that all participants received consistent guidance on proper technique and timing.

In addition, a video showing how to perform the 4‐7‐8 breathing exercise technique was sent as a text message to their cell phones. During one 4‐7‐8 breathing control cycle, the following steps were taken: (1) make a whoosh sound as you fully exhale; (2) shut your lips and take a calm breath through your nose while counting to four; (3) hold your breath while counting to seven; and (4) exhale completely through your mouth, counting to eight (Weil 2014). During the session, researchers checked whether the patients did the exercise correctly and provided feedback.

The patients were asked to perform these exercises twice a day (four cycles in the morning and four cycles in the evening) in a calm environment and in a sitting position for 6 weeks. To monitor adherence, the patients were expected to answer the questionnaire “Have you completed your breathing exercises, 4 cycles in the morning and 4 cycles in the evening?” via a WhatsApp message every day to confirm that participants were following the prescribed exercise routine. In addition, G.K. and G.S.U. reached the patients by phone every week and monitored whether they performed the exercises regularly. Any deviations from the prescribed regimen were noted and addressed with participants during follow‐up consultations.

The questionnaires were administered by S.B. and A.O. twice in total, before and on the day the 6‐week program was completed in the clinics for the experimental group. The patients in the control group were given informative session on tinnitus with the same procedure for the experimental group by researchers G.K. and G.S.U. for only 1 h. S.B. and A.O. administered the questionnaires twice, before and 6 weeks after the informative session in the clinics for the control group.

### 2.8 Data Collection Procedure

#### 2.8.1 Visual Analog Scale (VAS)

Patients were asked to assess the degree of distress and annoyance associated with their tinnitus, as well as its severity, using a VAS from 0 to 10, both before and after the application. An increase in these areas is shown by an increase in the score.

#### 2.8.2 Tinnitus Handicap Inventory (THI)

The THI assesses the catastrophic, emotional, and functional impacts of tinnitus and measures its effects on patients' daily functioning. The THI consists of 25 items (Aksoy et al. 2007). In the inventory, there are 25 items consisting of three options as “yes,” “no,” and “sometimes.” A “yes” answer is worth 4 points and a “no” answer is worth 0 points. A maximum score of 100 points can be obtained from the THI. The higher the score, the greater the perception of the tinnitus as a handicap (Aksoy et al. 2007).

#### 2.8.3 Insomnia Severity Index (ISI)

Sleep quality of the patients was assessed with the ISI. It is a questionnaire that evaluates the difficulties experienced in transitioning to and maintaining sleep, the level of stress caused by sleep problems, and impairments in daily functions, and thus determines the level of insomnia. It consists of 7 questions in total. A higher score indicates more insomnia symptoms (Boysan et al. 2010).

#### 2.8.4 Perceived Stress Scale‐10 (PSS‐10)

The PSS‐10 was administered to assess the stress perceived by tinnitus patients (Eskin et al. 2013). It was developed to measure the extent to which situations in one's life are considered stressful. It consists of 10 questions. The scores of PSS‐10 vary between 0 and 40. Greater perceived stress is indicated by higher scores (Eskin et al. 2013).

#### 2.8.5 Trait Anxiety Scale (TAS)

The trait anxiety subscale of the State‐Trait Anxiety Inventory was used to assess the level of anxiety in those with tinnitus. The TAS is a 4‐point Likert‐type scale consisting of 20 items that aims to determine how the individual feels regardless of the situation and conditions in which they find themselves. A score between 20 and 80 is obtained from this scale. Higher scores indicate a higher level of anxiety (Öner and Lecompte 1998).

#### 2.8.6 Statistical Analysis

The SPSS (Statistical Program in Social Sciences) 26 program was used to analyze the data. Number, percentage, mean, standard deviation, median, and min–max were calculated for descriptive statistics. To ascertain if the dataset adhered to a normal distribution, the Shapiro–Wilk test was used. Since the data distribution was not normal, nonparametric tests were used.

To examine the association between categorical data, the Pearson chi‐square test was used. For dependent groups, the Wilcoxon sign test was employed, and for independent groups, the Mann–Whitney *U* test.

## 3 Results

A total of 48 participants, 23 (13 women and 10 men) in the experimental group and 25 (12 women and 13 men) in the control group, took part in our study. The mean age of the experimental group was 43.69 ± 11.14 (25–65), while the mean age of the control group was 49.00 ± 9.60 (25–62). The ages of the participants did not show a statistically significant difference between the groups (*p* = 0.088). Gender distribution also did not differ between the groups (*p* = 0.555).

The mean tinnitus duration of the experimental group was 70.96 ± 13.65 months, and the mean tinnitus duration of the control group was 76.04 ± 15.34 months. There was no statistically significant difference in the tinnitus duration among groups (*p* = 0.764).

Nine participants felt tinnitus in the left ear, five in the right ear, and nine bilaterally in the experimental group. Fourteen participants felt tinnitus bilaterally, four in the left ear, and seven in the right ear in the control group. There was no difference between the groups in terms of tinnitus side (*p* = 0.195).

Descriptive statistical values of VAS scores for the groups and comparison of the within‐ and between‐groups' pre‐ and post‐application VAS scores are given in Table 1.

**TABLE 1:** The relationship between pre‐ and posttreatment VAS scores within‐ and between‐groups.

Tinnitus	Application	Experimental		Control		*p*	*d*
		Mean ± SD	*M*(Min–Max)	Mean ± SD	*M*(Min–Max)		
Severity	Pre	6.52 ± 1.78	6(3–10)	5.48 ± 2.82	5(2–10)	0.131m	0.28
	Post	4.04 ± 1.49	4(1–7)	5.60 ± 2.33	5(2–10)	0.025m	0.50
*p*		0.000w		0.499w			
*d*		0.92		−0.62			
Annoyance	Pre	6.30 ± 1.82	6(3–10)	6.00 ± 3.21	5(1–10)	0.609m	0.14
	Post	4.65 ± 2.52	5(0–10)	6.64 ± 2.39	7(3–10)	0.009m	0.40
*p*		0.001w		0.104w			
*d*		0.94		−0.38			
Distress	Pre	6.09 ± 2.37	6(0–10)	6.00 ± 3.43	5(0–10)	0.983m	0.07
	Post	3.65 ± 2.57	3(0–9)	6.16 ± 2.62	7(1–10)	0.002m	0.47
*p*		0.001w		0.761w			
*d*		0.81		−0.07			

Abbreviations: *d*, Cohen's *d*; m, Mann–Whitney *U* test; *M*, median; sd, standard deviation; w, Wilcoxon test.

Before the application, there was no statistically significant difference in the VAS scores between the groups (*p* > 0.05). Following the application, there was a significant difference in the experimental group's and the control group's VAS scores for these three categories (*p* < 0.05). All VAS scores of the experimental group were significantly lower than the control group (*p* < 0.05) (Table 1).

There was a statistically significant difference in all VAS scores of the experimental group before and after the application (*p* < 0.05). There was no statistically significant difference between all VAS scores of the control group before and after the informative session (*p* > 0.05) (Table 1).

Descriptive statistical values of the THI, PSS‐10, ISI, and TAS scores for the groups and comparisons of the questionnaire scores between and within groups before and after the application are shown in Table 2.

**TABLE 2:** The relationship between pre‐ and post‐intervention questionnaire scores within‐ and between‐groups.

Variables	Application	Experimental		Control		*p*	*d*
		Mean ± sd	*M*(Min–Max)	Mean ± sd	*M*(Min–Max)		
THI	Pre	39.04 ± 19.89	38(10–76)	45.84 ± 25.16	44(10–88)	0.426m	0.06
	Post	23.04 ± 19.71	18(0–76)	45.36 ± 26.39	42(4–94)	0.003m	0.44
*p*		0.001w		0.686w			
*d*		0.82		0.22			
PSS‐10	Pre	14.26 ± 6.41	15(0–29)	17.84 ± 6.09	17(3–30)	0.067m	0.29
	Post	11.61 ± 6.27	12(1–24)	15.52 ± 5.25	17(3–23)	0.007m	0.39
*p*		0.017w		0.076w			
*d*		0.61		0.21			
ISI	Pre	8.39 ± 5.55	9(1–18)	11.92 ± 6.28	11(0–21)	0.057m	0.27
	Post	4.35 ± 4.27	4(0–15)	12.28 ± 6.88	11(0–26)	0.000m	0.64
*p*		0.000w		0.819w			
*d*		0.92		0.13			
TAS	Pre	38.87 ± 7.32	38(25–60)	45.32 ± 7.04	45(30–55)	0.003m	0.44
	Post	34.22 ± 6.99	32(24–49)	43.96 ± 8.08	44(29–59)	0.000m	0.60
*p*		0.000w		0.194w			
*d*		0.88		0.35			

Abbreviations: *d*, Cohen's *d*; m, Mann–Whitney *U* test; *M*, median; sd, standard deviation; w, Wilcoxon test.

Before the application, there was no statistically significant difference in the THI, PSS‐10, or ISI scores between the groups (*p* > 0.05). On the other hand, there was a significant difference in the TAS scores between groups (*p* < 0.05). Following the application, THI, PSS‐10, ISI, and TAS scores showed a statistically significant difference between the groups (*p* < 0.05). After application, all questionnaire scores were significantly reduced in the experimental group compared to the control group (*p* < 0.05) (Table 2).

A statistically significant difference was found between the THI, PSS‐10, ISI, and TAS scores of the experimental group before and after the application (*p* < 0.05). The THI, PSS‐10, ISI, and TAS scores of the control group did not significantly change after the application (*p* > 0.05) (Table 2).

## 4 Discussion

To our knowledge, this is the first study to evaluate the effect of the 4‐7‐8 breathing technique on subjective perception of handicap caused by chronic tinnitus in daily life, tinnitus severity, annoyance, and distress caused by tinnitus, psychological factors, and sleep quality.

In terms of demographic information and tinnitus‐related features (duration and side), groups in our study did not differ significantly. The fact that there was no difference across the groups suggests that all of them shared the common characteristics (Aktaş and İlgin 2023). Aktaş and İlgin (2023) emphasized that the similarity of the groups in the evaluation of breathing exercise techniques is important for exercise reliability.

In our study, the pre‐intervention VAS scores and all scale scores of both groups were similar, and all groups showed no statistically significant difference, with the exception of the TAS scores. Aktaş and İlgin (2023) have suggested that obtaining similar results before any application increases the strength and reliability of the study. Pre‐intervention TAS scores for the control group were 45.32 ± 7.04 and 38.87 ± 7.32 for the experimental group. Although there was no dramatic difference between the questionnaire scores of the two groups, the small sample size of the study might be the cause of the statistically significant difference.

The body enters a state of deep relaxation through breathing techniques. Breathing slowly for a period enables the body to reabsorb oxygen. The 4‐7‐8 breathing method aids in the removal of carbon dioxide (CO_2_) and supplies organs and tissues with the oxygen they require (Yackle et al. 2017). In particular, deep and slow breathing raises parasympathetic activity, which informs the brain to calm down the body and regulates the body's response to anxiety (Vierra et al. 2022). Therefore, parasympathetic activation counteracts the effects of the sympathetic response, restoring balance to the autonomic nervous system (ANS) and reducing adrenaline levels. Breathwork like the 4‐7‐8 technique helps reverse the negative shifts in emotions, thoughts, behaviors, and physiological responses that arise during stressful situations. By activating the parasympathetic response, breathwork guides the mind and body back to a state of recovery and relaxation. This relaxation response counteracts the harmful effects of the “fight‐or‐flight” state (Shaw‐Metz 2023). Due to these effects, a reduction in anxiety levels is expected (Aktaş and İlgin 2023). Bhramari pranayama is a “Yogic” method that Pandey et al. used. It consists of a calming posture combined with the pushing of closed eyelids while simultaneously creating a humming sound that is comparable to tinnitus. The use of the Bhramari pranayama technique decreased annoyance, anxiety, and depression associated with tinnitus (Pandey et al. 2010). The authors highlighted that the humming functions as a self‐generated noise source and a calming strategy, and that this beneficial alteration may be the consequence of stimulating the parasympathetic nervous system (Pandey et al. 2010). In our study, following the application, there was a noticeable decrease in the experimental group's anxiety level. Moreover, a significant difference was obtained between groups. The control group's post‐application scores showed no significant differences. Köksoy et al. applied a four‐stage yoga exercise routine including pranayama and meditation, relaxation for 12 weeks in 12 tinnitus patients. They found a significant decrease in tinnitus handicap, severity, and stress scores. They proposed that yoga can lessen stress by regulating autonomic nerve activity with deep breathing and relaxing body muscles with certain positions (Köksoy et al. 2018). According to the results of Apoorva et al.'s (2023) narrative review study, it was suggested that yoga can be used as an effective strategy to overcome the psychosocial aspects of COVID‐19 in tinnitus patients. It was also emphasized that yoga has been documented to reduce the activity of the sympathetic nervous system by stimulating the parasympathetic nervous system, thus reducing stress and anxiety. Arif et al. (2017) applied mindfulness meditation to a group of tinnitus patients and relaxation therapy to a group of patients with tinnitus. In both groups, a significant decrease was found in tinnitus severity, loudness, distress, anxiety, and depression levels after the interventions. The HPA axis is stimulated during acute stress, and therefore, the stress hormone stimulates the nervous system to trigger a sympathetic response known as the “fight‐or‐flight” reaction (Shaw‐Metz 2023). Both stress responses and tinnitus share interactions with the HPA axis and ANS (Patil et al. 2023). Engaging in diaphragmatic breathwork like 4‐7‐8 technique during periods of high stress has been demonstrated to counteract sympathetic responses, reestablish parasympathetic relaxation in both mind and body, and enhance overall well‐being and equilibrium of physical and mental states (Shaw‐Metz 2023). In our study, the 4‐7‐8 breathwork provided a significant decrease in perceived stress, tinnitus handicap, tinnitus severity, and annoyance.

Further research in humans is necessary to elucidate how controlled breathing techniques influence brain networks related to emotional regulation and mood. Therefore, we suggest for future research to explore the potential mechanisms underlying the 4‐7‐8 breathing technique's effects on tinnitus more thoroughly, such as neuroimaging studies to assess changes in brain activity and autonomic function during and after the use of the 4‐7‐8 technique in individuals with tinnitus.

In our study, the experimental group's post‐intervention ratings were considerably lower than those of the group that had just received 1 h of informative session on tinnitus. No significant difference was found in all scores of the control group after informative session. Accordingly, a single informative session about tinnitus may not be sufficient to improve tinnitus‐related variables and psychological factors. Cima et al. (2012) found a substantial improvement in quality of life and a significant reduction in tinnitus severity in patients receiving customized therapy for tinnitus based on cognitive behavioral therapy compared to patients receiving usual care for tinnitus. Gilles et al. reported that only the tinnitus function index scores of patients who received a single 3‐h group psychoeducational tinnitus counseling session showed a significant decrease in the 6‐month follow‐up compared to the pretraining period. On the other hand, similar to our study, no significant change was found in VAS tinnitus severity, anxiety, and depression scores. The authors suggested that short‐term psychoeducational counseling for tinnitus patients may be a valid alternative to different tinnitus therapy programs (Gilles et al. 2022). In our study, the short duration of the tinnitus informative session (1 h) and the lack of long‐term follow‐up may have been the reason why we did not see a change in scores.

Niedziałek et al. found improvement in tinnitus perception, sleep, and quality of life in tinnitus patients following a 12‐week Ashtanga yoga course. Moreover, they used magnetic resonance imaging to detect a decrease in gray matter in the subparietal sulcus. They suggested that this result was indicative of an increased emotional calmness following yoga instruction. They emphasized that these changes may also lead to a general enhancement of patients' perception of tinnitus (Niedziałek et al. 2019). A significant decrease in the insomnia severity was obtained after the application of the 4‐7‐8 breathing exercise method in our study. Following the informative session, the insomnia severity levels of the control group did not significantly decrease. The experimental group's scores for the severity of insomnia were noticeably lower compared to those of the control group.

In our study, the 4‐7‐8 technique was applied twice a day, four cycles in the morning and four cycles in the evening, and lasted a total of 6 weeks. It has been suggested that this technique can be performed several times per day for no more than four cycles at a time until greater comfort levels are achieved (Shaw‐Metz 2023). In this sense, our study is parallel to this suggestion, and there is no consensus in the literature on how long this intervention should last. The duration of application of this technique applied to different groups in the literature varies (Vierra et al. 2022; Aktaş and İlgin 2023; Nielsen et al. 2023). In the future, studies comparing the effectiveness of different intervention durations in tinnitus patients can be planned.

Findings from the literature and our study show that yogic practices including pranayama technique and 4‐7‐8 breathing exercises can reduce anxiety, depression, stress, insomnia levels, and perception of tinnitus disability, severity, distress, and annoyance.

### 4.1 Limitations

Limitations of our study include a lack of long‐term follow‐up after therapy, short duration of therapy, small sample size, and lack of comparison with different breathing and yogic methods. In addition, the respiratory rate of the patients during exercise could not be objectively measured. We recommend future studies with larger sample sizes, long‐term therapy comparisons, and long‐term follow‐up after the end of therapy.

## 5 Conclusion

The 4‐7‐8 breathing exercise technique can be considered as a promising therapy method to reduce the severity of disability, annoyance, distress, insomnia, and psychological factors related to tinnitus. In light of the findings, we suggest that the 4‐7‐8 breathing exercise should be included in the routine of ENT physicians and audiologists in clinics as an alternative and simple but effective method in the therapy of tinnitus, and should be used as a complementary and supportive method.

## Author Contributions

**Gulce Kirazli**: conceptualization, methodology, data curation, investigation, writing–original draft, writing–review and editing, formal analysis, resources, project administration. **Suheda Baran**: methodology, writing–original draft, investigation, conceptualization, writing–review and editing, formal analysis, data curation, resources. **Gokce Saygi Uysal**: conceptualization, investigation, writing–original draft, writing–review and editing, methodology. **Aykut Ozdogan**: methodology, writing–original draft, formal analysis, investigation. **Serpil Mungan Durankaya**: writing–original draft, writing–review and editing, formal analysis, methodology. **Mehmet Fatih Ogut**: supervision, methodology, writing–original draft, writing–review and editing, formal analysis.

## Ethics Statement

Bakircay University's Ethics Committee for Non‐Interventional Clinical Research provided ethical permission for the study (Decision No.: 1018, Date: May 03, 2023).

## Consent

All participants signed the informed consent form.

## Conflicts of Interest

The authors declare no conflicts of interest.

## Data Availability

The analyzed datasets in the study are available from the corresponding author on reasonable request.
